# Pictorial Review on Imaging Findings in Cerebral CTP in Patients with Acute Stroke and Its Mimics: A Primer for General Radiologists

**DOI:** 10.3390/diagnostics13030447

**Published:** 2023-01-26

**Authors:** Benedikt Haggenmüller, Kornelia Kreiser, Nico Sollmann, Magdalena Huber, Daniel Vogele, Stefan A. Schmidt, Meinrad Beer, Bernd Schmitz, Yigit Ozpeynirci, Johannes Rosskopf, Christopher Kloth

**Affiliations:** 1 Department of Diagnostic and Interventional Radiology, Ulm University Medical Center, Albert-Einstein-Allee 23, 89081 Ulm, Germany; 2 Department of Radiology and Neuroradiology, RKU—Universitäts- und Rehabilitationskliniken Ulm, Oberer Eselsberg 45, 89081 Ulm, Germany; 3 Department of Neuroradiology, Bezirkskrankenhaus Günzburg, Lindenallee 2, 89312 Günzburg, Germany; 4 Institute of Neuroradiology, University Hospital, LMU Munich, Marchioninistr. 15, 81377 Munich, Germany

**Keywords:** computed tomography, CT perfusion, stroke

## Abstract

The imaging evaluation of computed tomography (CT), CT angiography (CTA), and CT perfusion (CTP) is of crucial importance in the setting of each emergency department for suspected cerebrovascular impairment. A fast and clear assignment of characteristic imaging findings of acute stroke and its differential diagnoses is essential for every radiologist. Different entities can mimic clinical signs of an acute stroke, thus the knowledge and fast identification of stroke mimics is important. A fast and clear assignment is necessary for a correct diagnosis and a rapid initiation of appropriate therapy. This pictorial review describes the most common imaging findings in CTP with clinical signs for acute stroke or other acute neurological disorders. The knowledge of these pictograms is therefore essential and should also be addressed in training and further education of radiologists.

## 1. Introduction

In the setting of an emergency department, the accurate imaging interpretation of cerebrovascular diseases and particularly ischemic stroke is of crucial importance. In addition to non-enhanced computed tomography (NECT), CT perfusion (CTP) and CT angiography (CTA), or the combination of both, are the most used imaging modalities in the setting of suspected acute stroke [1]. Magnetic resonance imaging (MRI) takes more time, is less cost-efficient, and is not applicable for every patient (e.g., not suited for patients with certain types of cardiac pacemakers or restless patients). In contrast, CT is the first-line imaging modality used in neurologic emergencies because of its wide availability and acquisition speed for acute intracranial disease (e.g., bleeding or stroke) [2,3]. Even in pediatric patients, automated perfusion imaging is feasible and can help to identify patients who would benefit from thrombectomy [4].

Specifically, CTP generates volumetric data for sufficient and fast differentiation between ischemic core and penumbra in the setting of an acute ischemic stroke [5,6]. It may also be helpful for rapid detection of any perfusion impairment stemming from the occlusion of peripheral arterial branches, which might be hard to detect on CTA [5]. Currently, more and more automated or even semiautomated postprocessing algorithms of CTP data enable fast analysis of the generated perfusion maps without dedicated technical or manual postprocessing [5,6]. Technical pitfalls including motion artifacts, poor signal-to-noise ratio, or a suboptimal arterial and venous input function are the most common problems [2,7]. Every radiologist must be aware of them and the limitations of this modality.

The Endovascular Therapy Following Imaging Evaluation for Ischemic Stroke 3 (DEFUSE 3) and Extending the Time for Thrombolysis in Emergency Neurological Deficits (EXTEND) trials have provided evidence that perfusion imaging can be applied to triage patients with acute ischemic stroke regarding reperfusion therapy, in addition to the conventionally used “time window” for treatment decision making [8,9]. However, different entities can mimic clinical signs of an acute ischemic stroke, thus the identification of those is important for every radiologist [10]. A fast and clear assignment of pathognomonic signs and imaging findings is the basis for a correct diagnosis. Awareness of the typical findings and pitfalls is therefore critical for radiologists to make accurate and timely decisions regarding an immediate treatment recommendation [2]. This pictorial review describes the most common imaging findings in CTP with clinical signs of acute neurological disorders.

## 2. Technique

Perfusion imaging aims to characterize microscopic flow at the capillary level. It can be performed by an intravenous injection of contrast medium that does not traverse the blood–brain barrier. Subsequently, rapid sequential imaging allows the visualization of the traverse effect of the contrast medium at the vascular system. The underlying mechanism is the central volume principle.

The increase in attenuation of brain parenchyma following administration of a contrast medium is compared to both a reference artery and a reference vein [11]. To obtain the arterial input function (AIF) and the venous output function (VOF), an arterial and a venous region of interest (ROI) must be defined, either manually by the user or with automated or semiautomated software. Large vessels orthogonal to the scanning plane—such as the anterior cerebral artery (ACA) and the dorsal part of the superior sagittal sinus—can help to minimize partial volume effects [12].

Cerebral blood volume (CBV), cerebral blood flow (CBF), mean transit time (MTT), and time to maximum (Tmax) are commonly evaluated. In detail, CBV represents the volume of blood within an imaging voxel including blood in the tissues and blood vessels (normal range 4–5 mL/100 g) [13]. In contrast, CBF represents the total volume of blood moving through a voxel per unit of time (normal range in gray matter, 50–60 mL/100 g/min, normal range in white matter 20–30 mL/100 g/min) [13]. In this regard, CBF is defined by the ratio of CBV divided by the MTT. 

The thresholds for penumbra and irreversible damage are dependent on the duration of hypoperfusion and the volume of the affected brain parenchyma. An early investigation using a series of positron emission tomography (PET) studies performed 5–18 h after stroke onset proposed a threshold for the penumbra of around 20 mL/100 g/min and documented that the extent of neurological recovery may be proportional to the volume of penumbra that eventually escaped infarction [14]. Within this time interval, the threshold for irreversible damage was around 8 mL/100 g/min for CBF [14]. 

Furthermore, Tmax is probably the most important parameter. It reflects the time delay between the contrast bolus arriving in the proximal large artery and each voxel of the brain parenchyma. It is calculated by deconvoluting the AIF [12]. It was used in landmark clinical trials for the definition of hypoperfusion of the brain parenchyma [8,15]. A threshold of Tmax > 6 s for predicting penumbral tissue has been established by several studies [16,17]. A target profile is used to determine patients who would benefit from thrombectomy that includes an ischemic core volume (CBF > 6 s) < 70 mL and severely delayed volume (Tmax > 10 s) < 100 mL [18]. If these criteria are met in a patient with vessel occlusion, a benefit from thrombectomy seems likely [18]. An additional overview of the parameters is given in Table 1.

The MTT is defined as the average (mean) transit time of all the molecules of a contrast medium in the brain volume and is measured in seconds. A minimum tissue slab coverage of 4–8 cm has been recommended by a large international consensus panel of stroke imaging experts [11,19].

Usually, CTP postprocessing uses either the maximum slope (MS) or a variant of the deconvolution (DC) approach for modeling of voxel-based time–attenuation curves [13,20,21]. The MS calculation is based on the area under the curve of each voxel influenced by the arterial flow and the tissue itself [1]. In this context, numerous factors can alter the temporal profile of arterial flow (e.g., low cardiac output, carotid artery stenosis, or injection-related characteristics) [1]. The DC models can be subdivided into singular value decomposition (SVD), delay- and dispersion-corrected singular value DC (dd-SVD), and Bayesian methodologies [1,22,23,24]. Specifically, dd-SVD or Bayesian-estimated models generate the arterial delay time in contrast to Tmax derived from the SVD model [24]. Use of different models can result in measurement variability of the core and penumbra based on the DC algorithm [24].

Advantages and disadvantages of the calculation models as well as the parameter cards differ between providers [25,26]. Perfusion maps can be manually generated in a short time at a workstation equipped with appropriate software; alternatively, semiautomated or fully automated approaches exist [11]. Besides others, a commonly used software in the clinical setup is the Rapid Processing of Perfusion and Diffusion (RAPID) software, which can be used for assessment of MRI-based perfusion and CTP scans [27]. The RAPID software has been used in several landmark studies and has been approved by the Food and Drug Administration (FDA) [27,28,29,30]. For the figures of this article, the perfusion maps were generated with the SyngoVia (version VB40B) application “*syngo* Volume Perfusion CT Neuro” (Siemens Healthineers, Erlangen, Germany; https://www.siemens-healthineers.com/de-ch/computed-tomography/options-upgrades/clinical-applications/syngo-volume-perfusion-ct-neuro, accessed on 25 January 2023). All scans and their evaluation for this work were performed with a Siemens Somatom Definition AS+ or a Siemens Definition Edge Plus CT scanner with a scan length up to 90 mm (Siemens Healthineers, Erlangen, Germany).

The CTP thresholds are necessary for determining normal unaffected tissue with unimpaired perfusion, infarct core, and penumbra, and to quantify the obtained results for different compartments [13]. Wintermark et al. showed that an elevated MTT of at least 145% compared with normal contralateral tissue is optimal as a means of defining tissue at risk [31]. Nevertheless, Kamalian et al. showed that the precise threshold values vary a lot, especially depending on the postprocessing technique [32]. The status of the literature has not yet been conclusively clarified.

Perfusion maps can be drawn from color-coded representations of CBF, CBV, and MTT values. Through the combination of these parameters, as well as other derived factors such as Tmax and TTP, detailed and spatially resolved perfusion metrics beyond those estimated directly from CTA can be obtained. Thereby, TTP could be a substitute for Tmax to avoid problems with AIF selection [33]. In acute ischemic stroke, the area of infarct is typically characterized by a prolonged MTT in combination with decreased CBV and CBF. According to the Monro–Kellie doctrine, every change in intracranial pressure (e.g., through stroke) is likely to result in changes in brain tissue mass, CBV, and CBF [34]. Areas of penumbra typically demonstrate prolonged MTT and TTP, because of the slower blood flow with normal to increased CBV [10,11,35]. In the context of persisting autoregulatory mechanisms, CBV can increase because of vasodilatation as an attempt to preserve constant CBF [10]. In demarked infarction areas this autoregulatory mechanism is lost. 

Ischemic stroke can cause vasogenic edema and a loss of grey and white matter differentiation (GWD) as a typical sign on NECT [2]. By semiautomated processes, even the semiquantitative Alberta Stroke Program Early CT Score (ASPECTS) can be calculated from NECT imaging, which represents a 10-point topographic CT scan score used in patients with middle cerebral artery (MCA) occlusion [36,37]. The ASPECT has already been shown to be relevant in the context of the decision for intravenous thrombolysis when the approach was still limited to a 3-h time window [36]. At that time, an ASPECTS ≤ 7 was defined as the threshold for patients being assumed to be functionally dependent [36,38]. 

Even extracerebral pathologies can cause changes in the intracerebral perfusion. For example, depending on the compensation mechanisms, chronic proximal stenosis of the internal carotid artery can result in prolonged MTT and TTP, and decreased CBV and CBF [39]. Alternatively, in the acute phase of ictal activity, an increase in CBF and CBV can be registered as a sign of vasodilatatory response to the increased metabolic demands [10]. Table 2 shows an overview of different entities and their characteristic imaging findings.

## 3. Imaging Findings

Typical imaging findings for CTP can be subdivided into those stemming from ischemic stroke and those resulting from non-stroke disorders. Non-stroke disorders can include different entities including metabolic (e.g., hypoglycemia), vascular (e.g., vasculitis or vasospasm), neoplasms (e.g., metastasis), infection (e.g., cerebral abscess), and inflammation (e.g., meningitis). Other important differential diagnoses for acute ischemic stroke are other neurological disorders such as migraine or epileptic seizures.

### 3.1. Acute Supratentorial Stroke

A current meta-analysis showed that CTP is more accurate than NECT and similar to CTA in detecting acute ischemic stroke [40]. In this meta-analysis, the pooled sensitivity of CTP in acute ischemic stroke regardless of specific territories was 82%, and the specificity was 96% [40]. In this context, the extent and size of infarct (e.g., measured by diffusion-weighted MRI) can influence both the sensitivity and specificity of CTP. Hana et al. found a higher sensitivity of CTP when the size of infarction was over 3 cm^2^ (90% vs. 29%, *p* < 0.001) [40,41]. According to this, the sensitivity of CTP was higher in patients with an average National Institutes of Health Stroke Scale (NIHSS) score of 8.3 than in a group which had an NIHSS score of 4.4 (100% vs. 42.6%) [40,42].

Tan et al. showed that CTA source images performed better than CTP in detecting the site of occlusion (sensitivity, 94.6%; specificity, 100.0% vs. sensitivity, 88.2%; specificity, 95.3%); however, perfusion maps were superior in predicting the anatomic distribution of the final infarct (sensitivity, 80.4%; specificity, 96.8% vs. sensitivity, 72.0%; specificity, 98.4%); though it has to be considered that 48 of the 55 patients in that study had an infarction within the MCA territory [35]. However, even though CTA is superior in detecting high degrees of cerebral arterial stenosis, CTP can provide a higher specificity in the detection of ischemia and infarct tissue of the brain, and the combination of both can lead to an accurate assessment for acute stroke [43]. In the direct comparison between MRI and CT imaging, there was a high specificity (91.8%) but poor sensitivity (40.0%) for MTT maps of CTP for predicting diffusion restriction of MRI regardless of the fluid attenuated inversion recovery (FLAIR) demarcation, as reported by Huisa et al. [44]. Nevertheless, regarding reperfusion treatment of acute stroke, MRI and combined CTA/CTP can lead to comparable treatment decisions [45].

However, CTP has limited diagnostic utility in acute lacunar strokes (approximately 50% false negative cases) and small cortical and subcortical strokes with a size of infarction under 3 cm^2^ [24,40]. Flat-detector CT (FD-CT) imaging in the angiography suite allowed rendering cone-beam CT images with additional value in interventional neuroradiology in acute stroke [46]. The collateral status in acute ischemic stroke patients is an important indicator for good outcome. In this context, perfusion imaging potentially allows for the simultaneous assessment of local perfusion and the collateral status [47]. 

In patients with acute ischemic stroke, leptomeningeal collateral blood flow potentially maintains blood supply to the ischemic region [47]. Accordingly, the collateral status may be associated with favorable outcome [47]. Collateral capacity can be assessed using several imaging modalities, including CTA that has been used to indirectly assess collateral status based on contrast filling in the arteries distal to the clot [47].

**Figure 1:**
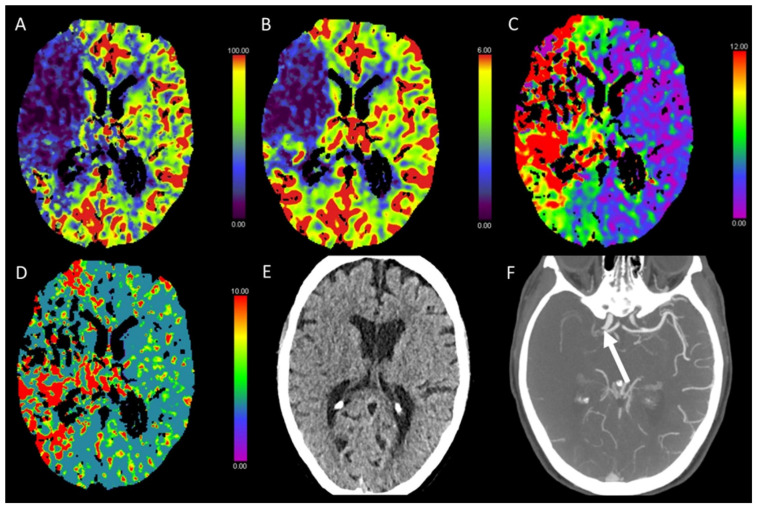
Color-coded maps generated from computed tomography perfusion (CTP) of the brain: (**A**) cerebral blood flow (CBF) [mL/100 g/min], (**B**) cerebral blood volume (CBV) [mL/100 g], (**C**) time to maximum (Tmax) [s], and (**D**) mean transit time (MTT) [s]. (**E**) Non-enhanced CT (NECT) and (**F**) arterial CT angiography (CTA). This patient underwent cardiac catheterization for closure of an atrial septal defect. Afterwards, the patient presented with a left-sided hemiparesis. NECT showed a slight reduction in the gray to white matter differentiation. In the territory of the middle cerebral artery (MCA) on the right side, CBF and CBV were reduced and Tmax and MTT were prolonged. CTA revealed an occlusion of the right distal internal carotid artery.

**Figure 2:**
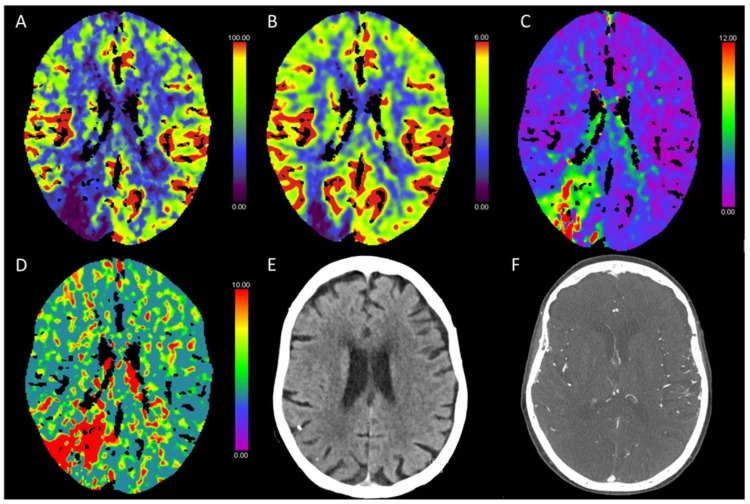
Color-coded maps generated from computed tomography perfusion (CTP) of the brain: (**A**) cerebral blood flow (CBF) [mL/100 g/min], (**B**) cerebral blood volume (CBV) [mL/100 g], (**C**) time to maximum (Tmax) [s], and (**D**) mean transit time (MTT) [s]. (**E**) Non-enhanced CT (NECT) and (**F**) arterial CT angiography (CTA). This patient presented with scotoma on the left side after transcatheter aortic valve implantation (TAVI). On NECT images, there was only a slight hypodense alteration of the cortex and a slight brain swelling in the right occipital cortex. CTP showed reduced CBF and CBV and prolonged Tmax and MTT in this area. No apparent vessel occlusion was found on CTA images.

**Figure 3:**
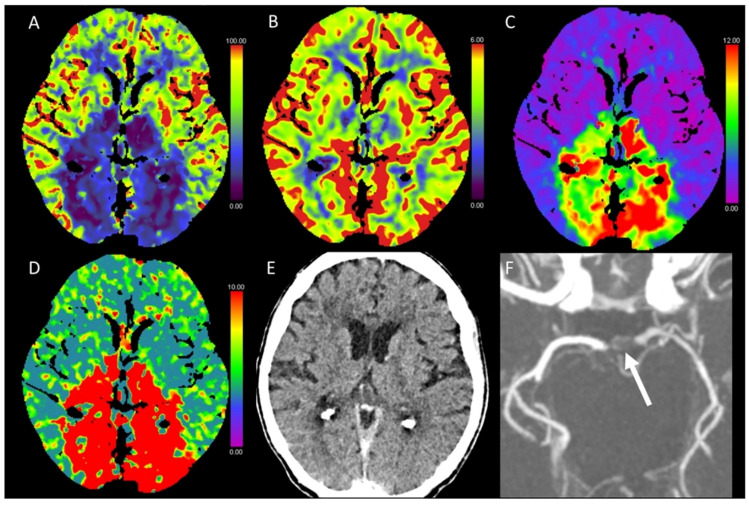
Color-coded maps generated from computed tomography perfusion (CTP) of the brain: (**A**) cerebral blood flow (CBF) [mL/100 g/min], (**B**) cerebral blood volume (CBV) [mL/100 g], (**C**) time to maximum (Tmax) [s], and (**D**) mean transit time (MTT) [s]. (**E**) Non-enhanced CT (NECT) and (**F**) arterial CT angiography (CTA) maximum intensity projection (MIP) images. This patient presented with anisocoria and became stuporous after transcatheter aortic valve implantation (TAVI). On NECT images, there were no detectable ischemic alterations of the brain parenchyma visible. Strongly reduced CBF and normal CBV resulting in strongly prolonged Tmax and MTT were found, consistent with an acute ischemic event and indicating a large penumbra without a circumscribed ischemic core. CTA revealed a thrombotic occlusion of the tip of the basilar artery.

**Figure 4:**
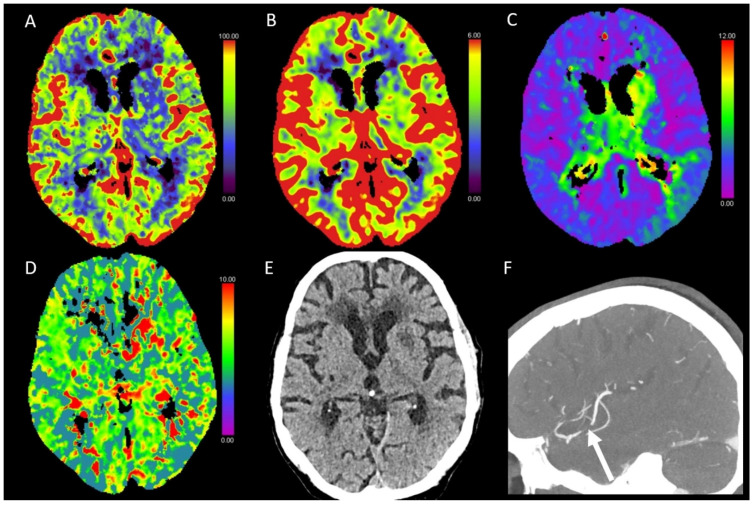
Color-coded maps generated from computed tomography perfusion (CTP) of the brain: (**A**) cerebral blood flow (CBF) [mL/100 g/min], (**B**) cerebral blood volume (CBV) [mL/100 g], (**C**) time to maximum (Tmax) [s], and (**D**) mean transit time (MTT) [s]. (**E**) Non-enhanced CT (NECT) and (**F**) arterial CT angiography (CTA). This patient presented with left-sided facial palsy, aphasia, and paresis of the right arm. NECT showed a beginning hypodense demarcation and a slight swelling in the area of the basal ganglia on the left side. Decreased CBF and only partly decreased CBV with analogous partly prolonged Tmax and MTT indicated an acute infarction. On CTA, there was an occlusion of the inferior trunk of the middle cerebral artery (MCA), which explained the slight prolongation of Tmax in the left temporal lobe.

**Figure 5:**
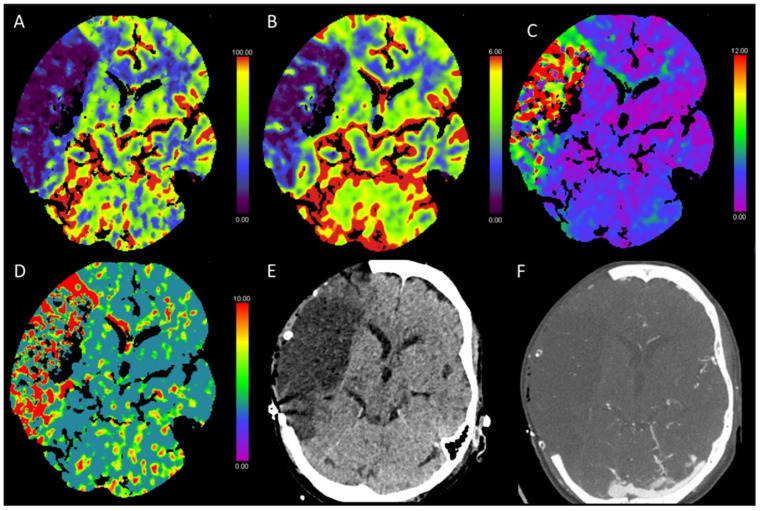
Color-coded maps generated from computed tomography perfusion (CTP) of the brain: (**A**) cerebral blood flow (CBF) [mL/100 g/min], (**B**) cerebral blood volume (CBV) [mL/100 g], (**C**) time to maximum (Tmax) [s], and (**D**) mean transit time (MTT) [s]. (**E**) Non-enhanced CT (NECT) and (**F**) arterial CT angiography (CTA). This study is a follow-up examination after right-sided hemicraniectomy for treatment of a malignant right middle cerebral artery (MCA) infarction. NECT showed hypodense alterations and swelling of the brain with a loss of the grey to white matter differentiation in the territory of the right middle cerebral artery (MCA), indicating widespread ischemic demarcation in this area. As expected, CBF and CBV were severely reduced and Tmax and MTT were prolonged. CTA showed reduced vessel contrast and vascular rarefaction in the infarction zone.

Examples for acute supratentorial stroke are given in Figure 1, Figure 2, Figure 3 and Figure 4. Figure 5 shows a follow-up examination of a malignant right MCA infarction.

### 3.2. Acute Posterior Fossa Infarcts 

Application of CTP is most often described for patients with supratentorial hemispheric ischemic stroke. Most commonly, CTP exams are performed with a focus on the level of the basal ganglia because it contains representative territories of the ACA, MCA, and posterior cerebral artery (PCA), whereas the areas above and below are only scanned with few thicker layers. The limited coverage of CTP scans could lead to neglecting posterior fossa infarcts, especially when the prior neurological examination and information do not necessarily pinpoint to this vascular territory. 

Different aspects must be considered in this context. First, posterior fossa infarcts can be small and may be undetected on CTP because of a small field of view focused on the basal ganglia, especially with older CT scanners [11]. Second, the beam hardening artifacts from the temporal bones could decrease the image quality, especially for the brain stem. Third, positioning of the field of view for CTP slices through the posterior fossa usually results in irradiation of the lenses and increases the deterministic risk of cataract. Nevertheless, prior work on posterior circulation strokes has reported a sensitivity of 76.6% and a specificity of 92.4% using CTP [48].

**Figure 6:**
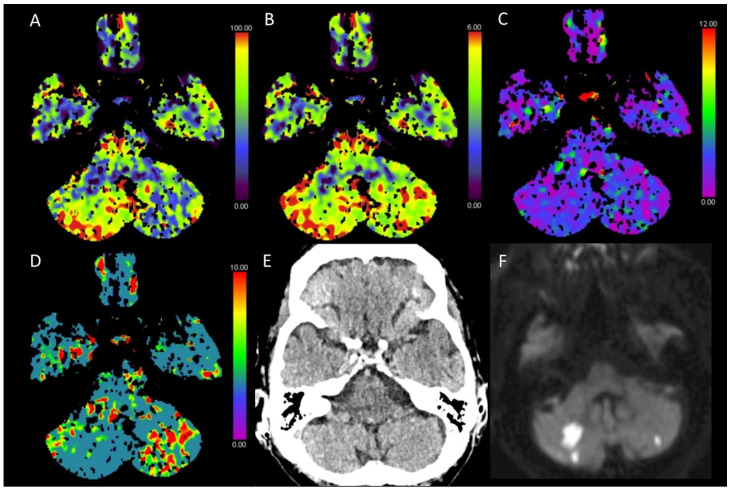
Color-coded maps generated from computed tomography perfusion (CTP) of the brain: (**A**) cerebral blood flow (CBF) [mL/100 g/min], (**B**) cerebral blood volume (CBV) [mL/100 g], (**C**) time to maximum (Tmax) [s], and (**D**) mean transit time (MTT) [s]. (**E**) Non-enhanced CT (NECT) and (**F**) diffusion-weighted magnetic resonance imaging (MRI) performed on the following day. This patient presented with myoclonus of the arms (especially on the left side), somnolence, and hypertension after transcatheter aortic valve implantation (TAVI). On NECT images, no distinct demarcations or brain swelling can be seen. On CTP maps, there was a reduced CBF and a normal CBV with a normal to slightly prolonged Tmax and a significantly prolonged MTT in the left cerebellar hemisphere, indicating an acute infarction within the posterior fossa. However, no vessel occlusion was found on CTA. Contrary to the findings from CTP, the MRI scan performed on the following day revealed small diffusion restrictions in the left cerebellar hemisphere and larger diffusion restrictions on the right side, indicative of bihemispheric acute ischemic insults.

**Figure 7:**
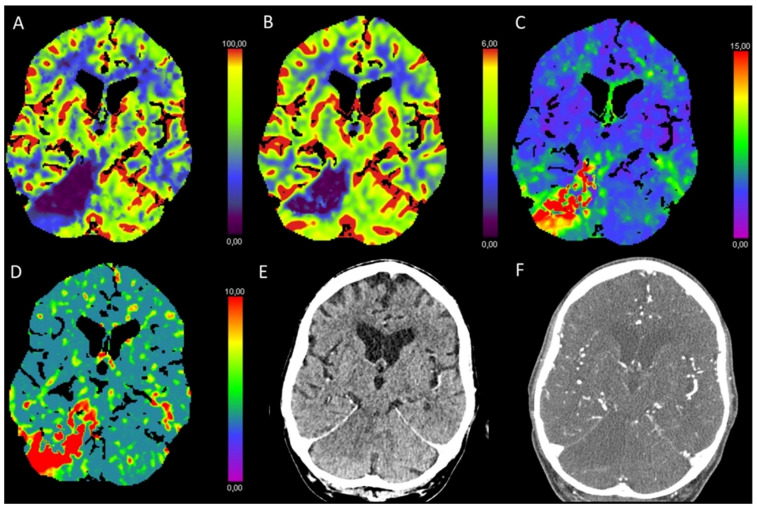
Color-coded maps generated from computed tomography perfusion (CTP) of the brain: (**A**) cerebral blood flow (CBF) [mL/100 g/min], (**B**) cerebral blood volume (CBV) [mL/100 g], (**C**) time to maximum (Tmax) [s], and (**D**) mean transit time (MTT) [s]. (**E**) Non-enhanced CT (NECT) and (**F**) arterial CT angiography (CTA). This patient presented with visual neglect, motor aphasia, and difficulty in swallowing after transcatheter aortic valve implantation (TAVI). On NECT images, there was a slight hypodense alteration and swelling of the superior right cerebellar hemisphere, indicating an acute infarction. On CTP maps, there was a reduced CBF and CBV with prolonged Tmax and MTT. CTA showed an absence of the right superior cerebellar artery, which is doubled on the left site. This suggested a complete occlusion, particularly given that aplasia of this vessel is very rare [51].

However, MRI is considered superior to CTP for acute posterior fossa infarcts because of the above-mentioned technical and anatomical limitations of CTP, especially regarding small infarcts [49]. Patients can present acutely with non-specific symptoms such as vertigo or nausea [50]. Examples for acute posterior fossa infarcts and the difficulties in detecting them by CTP are given in Figure 6 and Figure 7.

### 3.3. Global Hypoxic-Ischemic Injury/Brain Death

Cardiac arrest is a well-known cause of global brain ischemia. In this regard, CTP has been applied for the confirmation of global hypoxic-ischemic injury or following brain death by demonstrating the absence of cerebral parenchymal perfusion in several studies [52,53,54,55,56]. An increased intracranial pressure leads to a decrease, and finally an arrest in cerebral perfusion according to the Monro–Kellie hypothesis [55]. The capillary level is the first place in which cerebral circulatory arrest begins, which can be visualized by CTP. The absence of an opacification of the intracranial arteries and veins can be observed.

In this context, the earliest sign of cerebral circulatory arrest is a lack of opacification of the deep internal cerebral veins and the greater cerebral veins. The sensitivity of this finding in the diagnosis of cerebral circulatory arrest in CTA is 98–100% [57]. Missing contrast in the cerebral arterial circle is a less sensitive indicator of cerebral circulatory arrest, yet showing a sensitivity of 86–100% [57,58]. 

**Figure 8:**
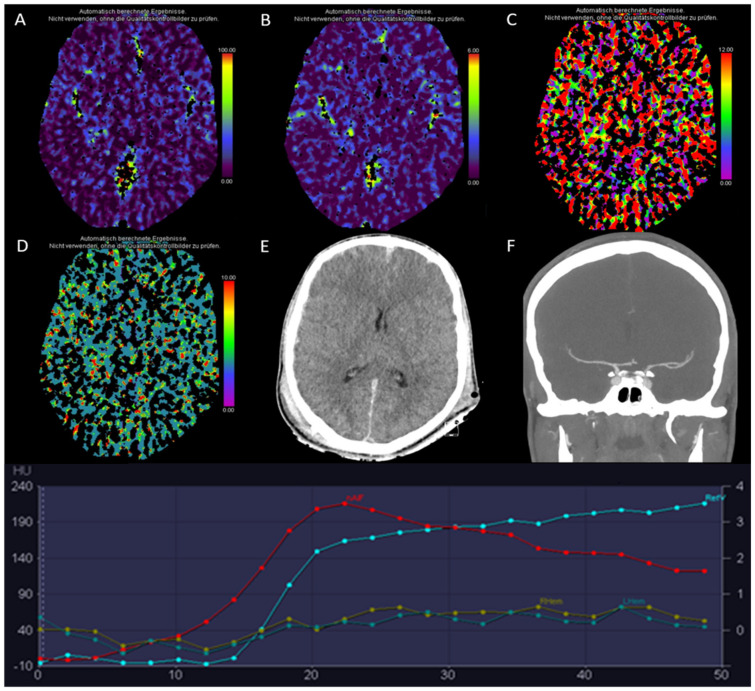
Color-coded maps generated from computed tomography perfusion (CTP) of the brain: (**A**) cerebral blood flow (CBF) [mL/100 g/min], (**B**) cerebral blood volume (CBV) [mL/100 g], (**C**) time to maximum (Tmax) [s], and (**D**) mean transit time (MTT) [s]. (**E**) Non-enhanced CT (NECT) and (**F**) arterial CT angiography (CTA). This patient was referred to the hospital after a traffic accident with a Glasgow Coma Scale (GCS) of 3. Besides skull fractures and a subarachnoid hemorrhage, NECT showed general brain swelling, loss of the grey to white matter differentiation, and severe reduction in the cerebrospinal fluid spaces due to a general cerebral edema. There was no detectable CBF or CBV according to CTP. On the CTA, the contrast in the intracranial arteries was still visible, but hardly recognizable in the veins. The time attenuation curve of the CTP showed that this was not the result of a technical failure or methodological issues: there was a regular increase in the arterial curve (red line) but a plateau in the venous curve (blue line), most likely due to the lack of parenchymal brain perfusion (right hemisphere: bright green line; left hemisphere: dark green line).

In the literature, conflicting results were described in up to 15% between CTA and CTP, with CTA sometimes showing some persistent intracranial flow while CTP shows a complete absence in cerebral perfusion [55,59]. An example is provided by Figure 8. 

A recommendation for a combination of CTP and CTA allowed reducing the number of false-negative results, especially regarding CTA alone [55]. Contrast stasis may affect the ROI measurements of CBF and CBV maps, and all quantitative analyses should be evaluated carefully.

### 3.4. Variation in Cerebrovascular Anatomy

**Figure 9:**
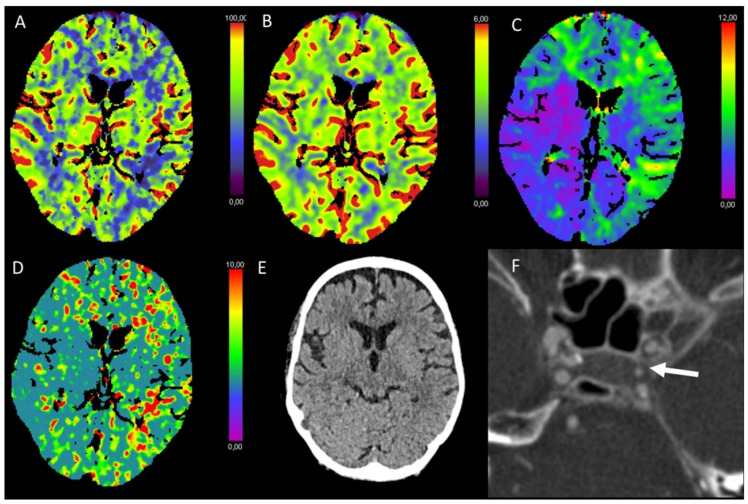
Color-coded maps generated from computed tomography perfusion (CTP) of the brain: (**A**) cerebral blood flow (CBF) [mL/100 g/min], (**B**) cerebral blood volume (CBV) [mL/100 g], (**C**) time to maximum (Tmax) [s], and (**D**) mean transit time (MTT) [s]. (**E**) Non-enhanced CT (NECT) and (**F**) arterial CT angiography (CTA). Slightly reduced CBF, regular CBV, and prolonged Tmax and MTT in the left hemisphere and the right frontal lobe suggested a delayed blood supply of this area. There were no ischemic demarcations visible on NECT. High-grade stenosis of the left internal carotid artery in the cavernous segment on CTA (arrow) and an aplastic A1 segment of the anterior cerebral artery (ACA) were detected. This patient presented with hemiparesis on the right side for several hours.

The circulus arteriosus Willisii regularly shows anatomic variations, which must be considered during imaging interpretation. Variations of the posterior communicating artery (PCA) can lead to asymmetry of hemodynamic parameters derived from perfusion imaging [11]. A correlation with the individual anatomic considerations on CTA is mandatory in each case. In particular, it can be problematic if a fetal PCA is present on one side, which can lead to asymmetry. Extracranial carotid and proximal intracranial carotid artery stenosis could cause hemodynamically prolonged MTT values in the territory supplied by the stenotic artery, even without brain ischemia [11]. Figure 9 shows an example of a variation in cerebrovascular anatomy.

### 3.5. Hypotensive Cerebral Infarction (HCI) with Watershed Infarcts/Border Zones

Border zone infarctions represent 10%–12% of all infarcts and are an important subset of ischemic lesions, which can oftentimes be identified or better characterized by their typical locations [60]. A reduction in the cerebral perfusion pressure caused by vessel occlusion, stenosis, or drastic reduced cardiac output causes such border-zone infarctions [31,61]. Two subtypes can be differentiated: border-zone infarctions at the cortex at the gray-white matter junctions, typically between major arterial territories at the confluence of two distal arterial territories, and infarction in deep white matter between perforating arteries. 

**Figure 10:**
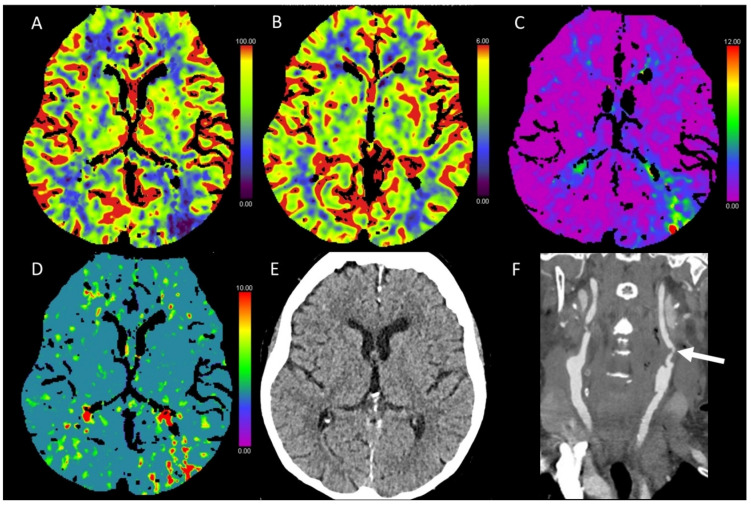
Color-coded maps generated from computed tomography perfusion (CTP) of the brain: (**A**) cerebral blood flow (CBF) [mL/100 g/min], (**B**) cerebral blood volume (CBV) [mL/100 g], (**C**) time to maximum (Tmax) [s], and (**D**) mean transit time (MTT) [s]. (**E**) Non-enhanced CT (NECT) and (**F**) arterial CT angiography (CTA). This patient underwent carotid thromboendarterectomy on the left side and presented with new paresis of the right arm. On NECT, there were no changes detectable. CTP maps showed decreased CBF and only slightly decreased CBV with prolonged Tmax and MTT in the parieto-occipital border zone of the left hemisphere, indicating perfusion restriction in the watershed zone. On CTA, there were thrombotic deposits in the carotid artery (white arrow) detected within the surgical site, leading to a stenosis of approximately 50%. Therefore, and due to the clinical finding of paresis, the patient underwent an immediate revision surgery with replacement of the vessel patch.

On CTP maps, typically a slight or even marked increase in CBV is registered in the impaired territory with reduced or maintained CBF as an indirect sign of flow compensation through the collateral circulation. The perfusion disturbances are most easily detected on the MTT maps [61]. An example is shown in Figure 10.

### 3.6. Vasospasm

Cerebral vasospasm is a frequent complication in patients with traumatic or atraumatic subarachnoid hemorrhage [62,63]. Vasospasm can cause delayed cerebral ischemia (DCI) with associated neurological and cognitive disabilities [63]. Besides CTA and digital subtraction angiography, quantitative parameters derived from CTP could be sufficient for identification of vasospasms when perfusion maps are evaluated visually [64,65]. In this context, vasospasms typically lead to hypoperfusion patterns that are associated with an increase in MTT and TTP [66]. In contrast, CBF and CBV may be normal, increased, or decreased depending on the severity of the vasospasm [66]. The degree of vasospasm could correlate with the perfusion deficit: Voldby et al. found that severe diffuse vasospasm, defined as a reduction in arterial caliber exceeding 50%, was associated with significant reduction in regional CBF [67,68]. Hattingen et al. showed that regional CBV decreases with an increasing degree of vasospasm and correlates with a decrease in regional CBF [69]. Lacking increases in regional CBV in territories with vasospasm to maintain CBF suggest a loss of cerebral autoregulation [69]. 

**Figure 11:**
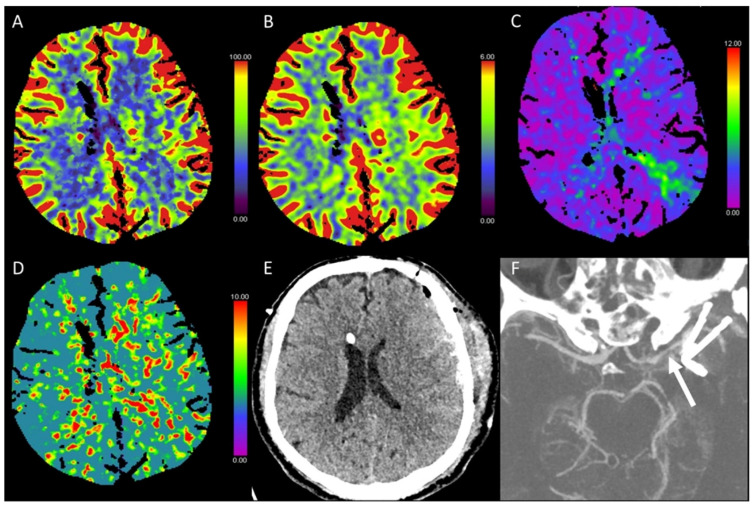
Color-coded maps generated from computed tomography perfusion (CTP) of the brain: (**A**) cerebral blood flow (CBF) [mL/100 g/min], (**B**) cerebral blood volume (CBV) [mL/100 g], (**C**) time to maximum (Tmax) [s], and (**D**) mean transit time (MTT) [s]. (**E**) Non-enhanced CT (NECT) and (**F**) arterial CT angiography (CTA) maximum intensity projection (MIP) images. This patient underwent surgery for clipping of a ruptured aneurysm of the proximal left middle cerebral artery (MCA), presenting with aphasia and somnolence after 6 days. The subarachnoid hemorrhage and the slight brain edema on the left side, seen on NECT, have already been known from earlier imaging exams. The CTA showed a narrowing of the left MCA (white arrow), indicative of vasospasms. CTP revealed a CBF/CBV mismatch of the left hemisphere with slightly prolonged Tmax in the border zones of the left hemisphere.

Overall, the highest diagnostic accuracy for vasospasm detection according to a meta-analysis is determined by both CTA and CTP in the setting of aneurysmal subarachnoid hemorrhage [70]. The overall range of reported sensitivity and specificity of CTA for detecting vasospasms was 63%–98% and 90%–98%, respectively [56]. Likewise, the overall range of reported sensitivity and specificity of CTP was 58%–95% and 86%–100%, respectively [70]. The sensitivity and specificity for a combination of CTA and CTP was not analyzed in that study [70]. An example of vasospasms is given in Figure 11. 

### 3.7. Migraine 

Both migraine and cluster headache affect the behavior of blood vessels as well as brain perfusion: cerebral arteries can change their diameter and influence brain perfusion directly [71]. Migraine can result in increased demand of oxygen and glucose with subsequent increases in focal brain perfusion.

In the acute aura phase of migraine and in the ictal phase of migraine without aura, patients can demonstrate hypoperfusion on CTP maps, thus an ischemic event could be erroneously assumed [13,71]. Olesen et al. described an initial reduction in CBF in the aura phase, followed by headache with a change in CBF from abnormally low to abnormally high values [71,72].

In a study by Floery et al., perfusion abnormalities were found in 70% of the patients [73]. They postulated that if migrainous aura is present, perfusion abnormalities are frequently observed. In all cases, patients were examined in the stage of migrainous aura, where neurologic deficits without headache were registered, and no hyperperfusion was detected [73]. Some published studies reported hyperperfusion; however, only during the headache stage at 6–24 h after the onset of symptoms [73,74,75].

### 3.8. Luxury Perfusion

Luxury perfusion is defined by the state of excessive brain blood flow in demand of the metabolic rate and oxygen demand of the brain tissue with a nearly normal or slight increase in CBV and CBF [76,77]. It is reported to occur in up to one third of patients by 48 h after ischemic stroke [76]. The incidence is higher in patients with intravenous or endovascular reperfusion treatment (48%) than in patients without (16%) [78]. Cerebral hyperperfusion is also known in patients after revascularization of stenotic or occluded cerebral vessels in chronic settings [79]. Luxury perfusion refers to a condition where CBF is in excess of oxygen demand in the situation of lost cerebrovascular autoregulation with non-nutritive flow to infarcted tissue. Another theory is based on the damage by free radicals, which can cause vasodilatation or increase the permeability of cerebral vessels. Third, a breakdown of baroreceptors in the carotid body due to angioplasty, stent placement or endarterectomy is discussed [79].

There are several complications related to cerebral hyperperfusion such as cerebral edema, seizure or hemorrhages, which can be detected clinically or by imaging [79]. As the luxury perfusion may be reversed at an early stage, an early diagnosis is important. Especially the edema is reversible, but if there is a hemorrhagic transformation the prognosis gets worse as up to 30% of patients remain at least partially disabled, and mortality rates are up to 50% [80,81]. Management of cranial hypertension includes blood pressure monitoring and antihypertensive agents [79]. The duration of antihypertensive treatment is unclear and there are also no definitive guidelines about the target blood pressure in these patients [79,82]. Anyway, cautious monitoring of blood pressure for at least 1 month is suggested in the literature for the restoration of cerebral autoregulation [79].

### 3.9. Veneous Thrombosis

Acute cerebral deep venous sinus thrombosis is a rare entity of stroke [83,84]. Establishing a correct diagnosis can be challenging due to its diverse and various clinical presentations [83]. It is more common in young women and can be associated with pregnancy or the use of hormonal contraceptives. Recently, also an association of cerebral venous sinus thrombosis with thrombocytopenia after Ad26.COV2.S vaccination has been suggested [85]. Venous thrombosis can cause venous congestion infarctions with or without hemorrhagic components [11]. In patients with cerebral venous thrombosis, who did not have evidence of stroke on follow-up imaging were found increased MTT with preserved CBV [83,86]. However, CBV could increase because of the inability of the venous system to drain the affected brain parenchyma. The most important CTP-derived imaging clues are the distribution of abnormal MTT and CBV with respect to venous vascular territories and the necessary comparison with the vein on CTA scans. 

Doege et al. used MRI-based perfusion imaging and compared perfusion deficits with changes on diffusion-weighted images [86]. Studies concluded that an increase in MTT in the absence of changes in CBV could be indicative of reversible parenchymal changes, analogous to the penumbra [83,86]. Gupta et al. showed that the range of values of relative CBV in the core and periphery of the lesions were similar [87]. Receiver operating curve analysis in their study proposed the optimal threshold values for relative CBF with > 60.5%, for relative CBV > 75.5%, and for rMTT < 148.5% [87].

Results by Mokin et al. suggested that CBV and CBF values might prove helpful in the differentiation between focal reversible changes, such as those from vasogenic edema, and irreversible changes due to infarction [83]. Specifically, CBV and CBF were reduced, and MTT was prolonged in both the core and periphery of lesions [83]. Studies using MRI have shown that focal changes caused by edema or infarction are found in only 25–50% of affected patients [83,88]. Both CT- and MRI-based venography exams were suitable for evaluation of the diagnosis of acute venous thrombosis [86,87]. Mimics of venous thrombosis especially on MRI can be flow gaps, anatomic variations, and large arachnoid granulations appearing as filling defects [60].

### 3.10. Postictal Stroke Mimics

**Figure 12:**
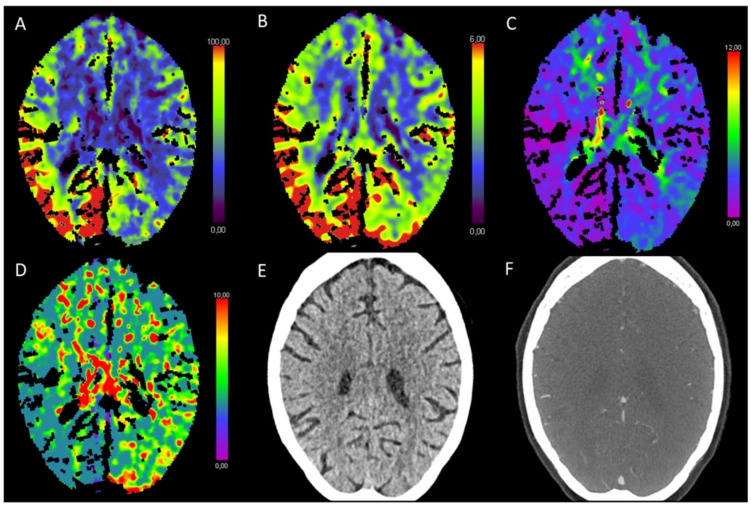
Color-coded maps generated from computed tomography perfusion (CTP) of the brain: (**A**) cerebral blood flow (CBF) [mL/100 g/min], (**B**) cerebral blood volume (CBV) [mL/100 g], (**C**) time to maximum (Tmax) [s], and (**D**) mean transit time (MTT) [s]. (**E**) Non-enhanced CT (NECT) and (**F**) arterial CT angiography (CTA). This patient with known epilepsy was referred to the hospital presenting with a hemiparesis on the left side. No changes were detected on NECT or CTA. Increased CBF and CBV with decreased Tmax and MTT in the right occipital and parietal lobes with the described clinical presentation suggested a convulsive event.

The clinical presentation of seizure is variable and can sometimes be difficult to distinguish from an ischemic stroke [11]. Different imaging findings can be recognized due to seizure activity: ictal, postictal, or interictal phases can be differentiated with varying and sometimes contrary results. Ictal CTP typically demonstrates decreased MTT, with increased CBF and CBV that are consistent with a hyperemic state [11,83]. However, in the postictal phase, the perfusion patterns can overlap with ischemic stroke including hypoperfusion [1]. Van Cauwenberge et al. showed that postictal patients can both show normal perfusion (64%), hypoperfusion (21%), or hyperperfusion (14%) on early CTP [89]. The results showed that CTP can differentiate ictal stroke mimics with hyperperfusion from acute ischemic stroke but could not allow to differentiate postictal patients who display perfusion patterns overlapping with ischemic stroke [89]. Typically, the perfusion alterations related to a seizure do not correspond to arterial vascular territories, so that a differentiation from stroke should be possible. Moreover, a study on nine patients showed that abnormalities related to seizure tend to involve the cortical gray matter, while mostly sparing the white matter [90]. An example of postictal stroke mimics is given in Figure 12.

### 3.11. Technical Pitfalls

**Figure 13:**
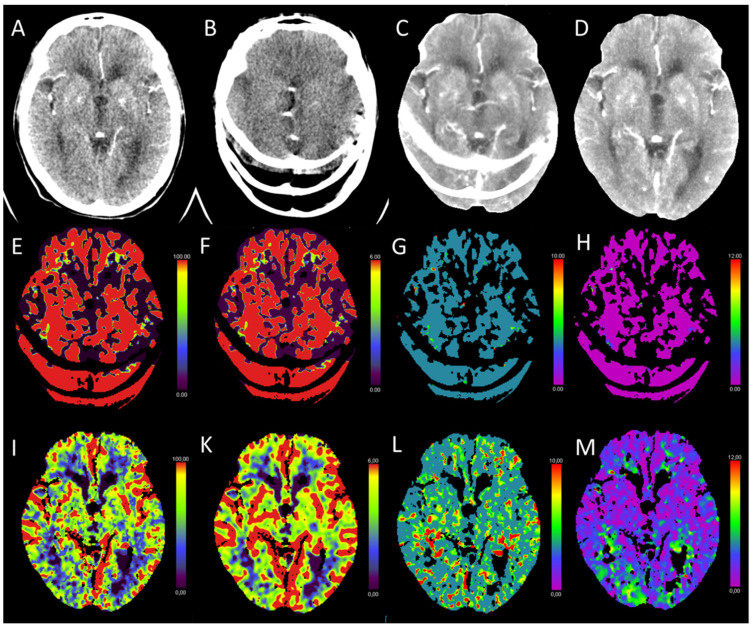
(**A**,**B**): computed tomography perfusion (CTP) of the brain at the same position at different time points. (**C**) maximum intensity projection (MIP) of the CTP generated by automated postprocessing software, (**D**) MIP of the CTP generated by manual postprocessing. (**E**–**H**): color-coded maps generated from CTP by automated postprocessing software: (**E**) cerebral blood flow (CBF) [mL/100 g/min], (**F**) cerebral blood volume (CBV) [mL/100 g], (**G**) mean transit time (MTT) [s], and **H**) time to maximum (Tmax) [s]. (**I**–**M**): color-coded maps generated from CTP by manual postprocessing: (**I**) CBF [mL/100 g/min], (**K**) CBV [mL/100 g], (**L**) MTT [s], and (**M**) Tmax [s]. Due to severe motion artifacts (**B**), the perfusion maps generated by automated postprocessing software algorithms were not usable for diagnostic purposes in this exemplary patient case. During the manual postprocessing steps, imaging time points with motion artifacts were excluded. The resulting manually generated perfusion maps (**I**–**M**) were usable and showed normal brain perfusion.

Motion artifacts, poor signal-to-noise ratio, or a suboptimal arterial and venous input function are described as the most common technical pitfalls for CTP [2,7]. Every radiologist must be aware of those issues and the limitations of the modality. Due to our clinical experience, most pitfalls are already solved by automated postprocessing software tools with features such as vessel recognition or motion correction. Nevertheless, sometimes an additional manual postprocessing can become necessary. Figure 13 outlines an example for a technical issue that has been resolved by post hoc manual corrections.

## 4. Conclusions

In emergency radiology, there are numerous diseases that can mimic acute stroke. The spectrum of diseases is wide, some conditions can be easily diagnosed, while some cases can be challenging. Memorable imaging findings of these entities from CTP are important. We presented several cases of acute stroke, its mimics, and their typical appearances. The knowledge of these pictograms is therefore essential for radiologists and should also be addressed in training and further education. This pictorial review should help the radiologist in the diagnosis and evaluation of cerebral pathologies. Radiologists should be aware of the different available imaging modalities, their benefits, limitations, and potential pitfalls.

## Figures and Tables

**Table 1:** Overview of different parameters in CTP.

Time to maximum	Tmax	The time to maximum reflects the time delay between the contrast bolus arriving in the proximal large artery and each voxel of the brain parenchyma. It is calculated by deconvoluting the arterial input function.
Time to peak	TTP	The time to peak reflects the time delay from the start of the scan until the maximum intensity of a contrast bolus arriving at each voxel of the brain parenchyma.
Mean transit time	MTT	The mean transit time represents the average time required for a contrast bolus to traverse the voxel.
Cerebral blood flow	CBF	The cerebral blood flow reflects the volume of blood flowing into a unit of brain tissue during a unit of time. $\mathrm{CBF}=\frac{\mathrm{CBV}}{\mathrm{MTT}}$
Cerebral blood volume	CBV	The cerebral blood volume reflects the fraction of a voxel that contains blood vessels (expressed as mL/100 g).

**Table 2:** Overview of different entities and their characteristic imaging findings. **↓** decreased, **↑** increased, **-** no changes.

Entity	CBV	CBF	MTT	TTP/Tmax
Acute stroke	**↓**	**↓/-**	**↑**	**↑**
Brain death	**↓↓**	**↓↓**	**↑↑**	**↑↑**
Vasospasm	**-/↓**	**-/↓**	**↑**	**↑**
Hypotensive cerebral infarction (HCI) with watershed infarcts/border zones	**↑**	**↓/-**	**↑**	**↑**
Migraine—aura phase		**↓**		
Seizure—ictal phase	**↑**	**↑**	**↓**	**↓**
—interictal phase	**↓**	**↓**	**↑**	
—postictal phase	**↓**	**↓**	**↑**	**↑**
Venous thrombosis—without venous ischemia or intracranial hemorrhage	**-/↑**	**-/↑**	**↑**	**↑**
Venous thrombosis—with venous ischemia and/or intracranial hemorrhage	**↓**	**↓**	**↑**	**↑**

## Data Availability

Not applicable.

## References

[1] Vagal A., Wintermark M., Nael K., Bivard A., Parsons M., Grossman A.W., Khatri P. (2019). Automated CT perfusion imaging for acute ischemic stroke: Pearls and pitfalls for real-world use. Neurology.

[2] Potter C.A., Vagal A.S., Goyal M., Nunez D.B., Leslie-Mazwi T.M., Lev M.H. (2019). CT for Treatment Selection in Acute Ischemic Stroke: A Code Stroke Primer. Radiographics.

[3] Tomandl B.F., Klotz E., Handschu R., Stemper B., Reinhardt F., Huk W.J., Eberhardt K.E., Fateh-Moghadam S. (2003). Comprehensive imaging of ischemic stroke with multisection CT. Radiographics.

[4] Lee S., Mlynash M., Christensen S., Jiang B., Wintermark M., StrÃ Ter R., Broocks G., Grams Austria A., Nikoubashman O., Morotti A. (2022). Hyperacute Perfusion Imaging Before Pediatric Thrombectomy: Analysis of the Save ChildS Study. Neurology.

[5] Hoeffner E.G., Case I., Jain R., Gujar S.K., Shah G.V., Deveikis J.P., Carlos R.C., Thompson B.G., Harrigan M.R., Mukherji S.K. (2004). Cerebral perfusion CT: Technique and clinical applications. Radiology.

[6] Srinivasan A., Goyal M., Al Azri F., Lum C. (2006). State-of-the-art imaging of acute stroke. Radiographics.

[7] Fahmi F., Beenen L.F.M., Streekstra G.J., Janssen N.Y., de Jong H.W., Riordan A., Roos Y.B., Majoie C.B., Vanbavel E., Marquering H.A. (2013). Head movement during CT brain perfusion acquisition of patients with suspected acute ischemic stroke. Eur. J. Radiol..

[8] Albers G.W., Marks M.P., Kemp S., Christensen S., Tsai J.P., Ortega-Gutierrez S., McTaggart R.A., Torbey M.T., Kim-Tenser M., Leslie-Mazwi T. (2018). DEFUSE 3 Investigators Thrombectomy for Stroke at 6 to 16 h with Selection by Perfusion Imaging. N. Engl. J. Med..

[9] Ma H., Campbell B.C.V., Parsons M.W., Churilov L., Levi C.R., Hsu C., Kleinig T.J., Wijeratne T., Curtze S., Dewey H.M. (2019). Thrombolysis Guided by Perfusion Imaging up to 9 h after Onset of Stroke. N. Engl. J. Med..

[10] Chiu A.H., Phillips T.J., Phatouros C.C., Singh T.P., Hankey G.J., Blacker D.J., McAuliffe W. (2016). CT perfusion in acute stroke calls: A pictorial review and differential diagnoses. J. Med. Imaging Radiat. Oncol..

[11] Keedy A., Soares B., Wintermark M. (2012). A pictorial essay of brain perfusion-CT: Not every abnormality is a stroke!. J. Neuroimaging.

[12] Krishnan P., Murphy A., Aviv R.I. (2017). CT-based Techniques for Brain Perfusion. Top. Magn. Reson. Imaging.

[13] Mangla R., Ekhom S., Jahromi B.S., Almast J., Mangla M., Westesson P. (2014). CT perfusion in acute stroke: Know the mimics, potential pitfalls, artifacts, and technical errors. Emerg. Radiol..

[14] Baron J.C. (2001). Perfusion thresholds in human cerebral ischemia: Historical perspective and therapeutic implications. Cerebrovasc. Dis..

[15] Nogueira R.G., Jadhav A.P., Haussen D.C., Bonafe A., Budzik R.F., Bhuva P., Yavagal D.R., Ribo M., Cognard C., Hanel R.A. (2018). DAWN Trial Investigators Thrombectomy 6 to 24 h after Stroke with a Mismatch between Deficit and Infarct. N. Engl. J. Med..

[16] Olivot J., Mlynash M., Thijs V.N., Kemp S., Lansberg M.G., Wechsler L., Bammer R., Marks M.P., Albers G.W. (2009). Optimal Tmax threshold for predicting penumbral tissue in acute stroke. Stroke.

[17] Campbell B.C.V., Christensen S., Levi C.R., Desmond P.M., Donnan G.A., Davis S.M., Parsons M.W. (2012). Comparison of computed tomography perfusion and magnetic resonance imaging perfusion-diffusion mismatch in ischemic stroke. Stroke.

[18] Laughlin B., Chan A., Tai W.A., Moftakhar P. (2019). RAPID automated CT perfusion in clinical practice. Pract. Neurol..

[19] Wintermark M., Albers G.W., Alexandrov A.V., Alger J.R., Bammer R., Baron J., Davis S., Demaerschalk B.M., Derdeyn C.P., Donnan G.A. (2008). Acute stroke imaging research roadmap. Stroke.

[20] Thaiss W.M., Haberland U., Kaufmann S., Spira D., Thomas C., Nikolaou K., Horger M., Sauter A.W. (2016). Iodine concentration as a perfusion surrogate marker in oncology: Further elucidation of the underlying mechanisms using Volume Perfusion CT with 80 kVp. Eur. Radiol..

[21] Thaiss W.M., Haberland U., Kaufmann S., Hepp T., Schulze M., Blum A.C., Ketelsen D., Nikolaou K., Horger M., Sauter A.W. (2019). Dose Optimization of Perfusion-derived Response Assessment in Hepatocellular Carcinoma Treated with Transarterial Chemoembolization: Comparison of Volume Perfusion CT and Iodine Concentration. Acad. Radiol..

[22] Konstas A.A., Goldmakher G.V., Lee T.Y., Lev M.H. (2009). Theoretic basis and technical implementations of CT perfusion in acute ischemic stroke, part 2: Technical implementations. AJNR Am. J. Neuroradiol..

[23] Bivard A., Levi C., Spratt N., Parsons M. (2013). Perfusion CT in acute stroke: A comprehensive analysis of infarct and penumbra. Radiology.

[24] Václavík D., Volný O., Cimflová P., Švub K., Dvorníková K., Bar M. (2022). The importance of CT perfusion for diagnosis and treatment of ischemic stroke in anterior circulation. J. Integr. Neurosci..

[25] Kamalian S., Kamalian S., Maas M.B., Goldmacher G.V., Payabvash S., Akbar A., Schaefer P.W., Furie K.L., Gonzalez R.G., Lev M.H. (2011). CT cerebral blood flow maps optimally correlate with admission diffusion-weighted imaging in acute stroke but thresholds vary by postprocessing platform. Stroke.

[26] Kudo K., Sasaki M., Yamada K., Momoshima S., Utsunomiya H., Shirato H., Ogasawara K. (2010). Differences in CT perfusion maps generated by different commercial software: Quantitative analysis by using identical source data of acute stroke patients. Radiology.

[27] Zhang Y., Song S., Li Z., Huang B., Geng Y., Zhang L. (2022). The Application of Software “Rapid Processing of Perfusion and Diffusion” in Acute Ischemic Stroke. Brain Sci..

[28] Lansberg M.G., Straka M., Kemp S., Mlynash M., Wechsler L.R., Jovin T.G., Wilder M.J., Lutsep H.L., Czartoski T.J., Bernstein R.A. (2012). DEFUSE 2 study investigators MRI profile and response to endovascular reperfusion after stroke (DEFUSE 2): A prospective cohort study. Lancet Neurol..

[29] Campbell B.C.V., Mitchell P.J., Yan B., Parsons M.W., Christensen S., Churilov L., Dowling R.J., Dewey H., Brooks M., Miteff F. (2014). EXTEND-IA investigators A multicenter, randomized, controlled study to investigate EXtending the time for Thrombolysis in Emergency Neurological Deficits with Intra-Arterial therapy (EXTEND-IA). Int. J. Stroke.

[30] Albers G.W., Lansberg M.G., Kemp S., Tsai J.P., Lavori P., Christensen S., Mlynash M., Kim S., Hamilton S., Yeatts S.D. (2017). A multicenter randomized controlled trial of endovascular therapy following imaging evaluation for ischemic stroke (DEFUSE 3). Int. J. Stroke.

[31] Wintermark M., Flanders A.E., Velthuis B., Meuli R., van Leeuwen M., Goldsher D., Pineda C., Serena J., van der Schaaf I., Waaijer A. (2006). Perfusion-CT assessment of infarct core and penumbra: Receiver operating characteristic curve analysis in 130 patients suspected of acute hemispheric stroke. Stroke.

[32] Kamalian S., Kamalian S., Konstas A.A., Maas M.B., Payabvash S., Pomerantz S.R., Schaefer P.W., Furie K.L., Gonzalez R.G., Lev M.H. (2012). CT perfusion mean transit time maps optimally distinguish benign oligemia from true “at-risk” ischemic penumbra, but thresholds vary by postprocessing technique. Ajnr Am. J. Neuroradiol..

[33] Wouters A., Christensen S., Straka M., Mlynash M., Liggins J., Bammer R., Thijs V., Lemmens R., Albers G.W., Lansberg M.G. (2017). A Comparison of Relative Time to Peak and Tmax for Mismatch-Based Patient Selection. Front. Neurol..

[34] Muizelaar J.P., Fatouros P.P., Schroder M.L. (1997). A new method for quantitative regional cerebral blood volume measurements using computed tomography. Stroke.

[35] Tan J.C., Dillon W.P., Liu S., Adler F., Smith W.S., Wintermark M. (2007). Systematic comparison of perfusion-CT and CT-angiography in acute stroke patients. Ann. Neurol..

[36] Barber P.A., Demchuk A.M., Zhang J., Buchan A.M. (2000). Validity and reliability of a quantitative computed tomography score in predicting outcome of hyperacute stroke before thrombolytic therapy. ASPECTS Study Group. Alberta Stroke Programme Early CT Score. Lancet.

[37] Pexman J.H., Barber P.A., Hill M.D., Sevick R.J., Demchuk A.M., Hudon M.E., Hu W.Y., Buchan A.M. (2001). Use of the Alberta Stroke Program Early CT Score (ASPECTS) for assessing CT scans in patients with acute stroke. AJNR Am. J. Neuroradiol..

[38] Hill M.D., Buchan A.M. (2005). Canadian Alteplase for Stroke Effectiveness Study (CASES) Investigators Thrombolysis for acute ischemic stroke: Results of the Canadian Alteplase for Stroke Effectiveness Study. CMAJ.

[39] Szarmach A., Halena G., Buczny J., Studniarek M., Markiet K., Szurowska E., Retkowski M., Piskunowicz M. (2011). Evaluation of changes in the parameters of brain tissue perfusion in multi-slice computed tomography in patients after carotid artery stenting. Pol. J. Radiol..

[40] Shen J., Li X., Li Y., Wu B. (2017). Comparative accuracy of CT perfusion in diagnosing acute ischemic stroke: A systematic review of 27 trials. PLoS ONE.

[41] Hana T., Iwama J., Yokosako S., Yoshimura C., Arai N., Kuroi Y., Koseki H., Akiyama M., Hirota K., Ohbuchi H. (2014). Sensitivity of CT perfusion for the diagnosis of cerebral infarction. J. Med. Investig..

[42] Eckert B., Kusel T., Leppien A., Michels P., Muller-Jensen A., Fiehler J. (2011). Clinical outcome and imaging follow-up in acute stroke patients with normal perfusion CT and normal CT angiography. Neuroradiology.

[43] Sabarudin A., Subramaniam C., Sun Z. (2014). Cerebral CT angiography and CT perfusion in acute stroke detection: A systematic review of diagnostic value. Quant. Imaging Med. Surg..

[44] Huisa B.N., Neil W.P., Schrader R., Maya M., Pereira B., Bruce N.T., Lyden P.D. (2014). Clinical use of computed tomographic perfusion for the diagnosis and prediction of lesion growth in acute ischemic stroke. J. Stroke Cerebrovasc. Dis..

[45] Wintermark M., Meuli R., Browaeys P., Reichhart M., Bogousslavsky J., Schnyder P., Michel P. (2007). Comparison of CT perfusion and angiography and MRI in selecting stroke patients for acute treatment. Neurology.

[46] van der Zijden T., Mondelaers A., Voormolen M., Menovsky T., Niekel M., Jardinet T., Van Thielen T., D’Archambeau O., Parizel P.M. (2022). Flat Detector CT with Cerebral Pooled Blood Volume Perfusion in the Angiography Suite: From Diagnostics to Treatment Monitoring. Diagnostics.

[47] Prasetya H., Tolhuisen M.L., Koopman M.S., Kappelhof M., Meijer F.J., Yo L.S., á Nijeholt G.J.L., van Zwam W.H., van der Lugt A., Roos Y.B. (2022). Value of CT Perfusion for Collateral Status Assessment in Patients with Acute Ischemic Stroke. Diagnostics.

[48] Van Der Hoeven E.J., Dankbaar J.W., Algra A., Vos J.A., Niesten J.M., Van Seeters T., Van Der Schaaf I.C., Schonewille W.J., Kappelle L.J., Velthuis B.K. (2015). DUST Investigators Additional diagnostic value of computed tomography perfusion for detection of acute ischemic stroke in the posterior circulation. Stroke.

[49] Hwang D.Y., Silva G.S., Furie K.L., Greer D.M. (2012). Comparative sensitivity of computed tomography vs. magnetic resonance imaging for detecting acute posterior fossa infarct. J. Emerg. Med..

[50] Ostman C., Garcia-Esperon C., Lillicrap T., Tomari S., Holliday E., Levi C., Bivard A., Parsons M.W., Spratt N.J. (2020). Multimodal Computed Tomography Increases the Detection of Posterior Fossa Strokes Compared to Brain Non-contrast Computed Tomography. Front. Neurol..

[51] Krzyżewski R.M., Stachura M.K., Stachura A.M., Rybus J., Tomaszewski K.A., Klimek-Piotrowska W., Brzegowy P., Urbanik A., Walocha J.A. (2014). Variations and morphometric analysis of the proximal segment of the superior cerebellar artery. Neurol. Neurochir. Pol..

[52] Escudero D., Otero J., Marqués L., Parra D., Gonzalo J.A., Albaiceta G.M., Cofiño L., Blanco A., Vega P., Murias E. (2009). Diagnosing brain death by CT perfusion and multislice CT angiography. Neurocrit. Care.

[53] Shankar J.J.S., Vandorpe R. (2013). CT Perfusion for Confirmation of Brain Death. Am. J. Neuroradiol..

[54] Bohatyrewicz R., Sawicki M., Walecka A., Walecki J., Rowinski O., Bohatyrewicz A., Kanski A., Czajkowski Z., Krzysztalowski A., Solek-Pastuszka J. (2010). Computed tomographic angiography and perfusion in the diagnosis of brain death. Transplant. Proc..

[55] Akdogan A.I., Pekcevik Y., Sahin H., Pekcevik R. (2021). Assessment of Cerebral Circulatory Arrest via CT Angiography and CT Perfusion in Brain Death Confirmation. Korean J. Radiol..

[56] MacDonald D., Stewart-Perrin B., Shankar J.J.S. (2022). The role of neuroimaging in the determination of brain death. Radiol. Bras.

[57] Sawicki M., Bohatyrewicz R., Walecka A., Sołek-Pastuszka J., Rowiński O., Walecki J. (2014). CT Angiography in the Diagnosis of Brain Death. Pol. J. Radiol..

[58] Frampas E., Videcoq M., de Kerviler E., Ricolfi F., Kuoch V., Mourey F., Tenaillon A., Dupas B. (2009). CT angiography for brain death diagnosis. AJNR Am. J. Neuroradiol..

[59] Kramer A.H., Roberts D.J. (2014). Computed tomography angiography in the diagnosis of brain death: A systematic review and meta-analysis. Neurocrit. Care.

[60] Krieger D.A., Dehkharghani S. (2015). Magnetic Resonance Imaging in Ischemic Stroke and Cerebral Venous Thrombosis. Top. Magn. Reson. Imaging.

[61] Siemund R., Cronqvist M., Andsberg G., Ramgren B., Knutsson L., Holtas S. (2009). Cerebral perfusion imaging in hemodynamic stroke: Be aware of the pattern. Interv. Neuroradiol..

[62] Kassell N.F., Sasaki T., Colohan A.R., Nazar G. (1985). Cerebral vasospasm following aneurysmal subarachnoid hemorrhage. Stroke.

[63] Othman A.E., Afat S., Nikoubashman O., Muller M., Schubert G.A., Bier G., Brockmann M.A., Wiesmann M., Brockmann C. (2016). Volume perfusion CT imaging of cerebral vasospasm: Diagnostic performance of different perfusion maps. Neuroradiology.

[64] Cremers C.H.P., van der Schaaf I.C., Wensink E., Greving J.P., Rinkel G.J.E., Velthuis B.K., Vergouwen M.D.I. (2014). CT perfusion and delayed cerebral ischemia in aneurysmal subarachnoid hemorrhage: A systematic review and meta-analysis. J. Cereb. Blood Flow Metab..

[65] van der Schaaf I., Wermer M.J., van der Graaf Y., Hoff R.G., Rinkel G.J.E., Velthuis B.K. (2006). CT after subarachnoid hemorrhage: Relation of cerebral perfusion to delayed cerebral ischemia. Neurology.

[66] Ritzenthaler T., Gobert F., Dailler F. (2019). “Vasospasm mimic” after aneurysmal subarachnoid hemorrhage. World Neurosurg..

[67] Voldby B., Enevoldsen E.M., Jensen F.T. (1985). Regional CBF, intraventricular pressure, and cerebral metabolism in patients with ruptured intracranial aneurysms. J. Neurosurg..

[68] Aralasmak A., Akyuz M., Ozkaynak C., Sindel T., Tuncer R. (2009). CT angiography and perfusion imaging in patients with subarachnoid hemorrhage: Correlation of vasospasm to perfusion abnormality. Neuroradiology.

[69] Hattingen E., Blasel S., Dettmann E., Vatter H., Pilatus U., Seifert V., Zanella F.E., Weidauer S. (2008). Perfusion-weighted MRI to evaluate cerebral autoregulation in aneurysmal subarachnoid haemorrhage. Neuroradiology.

[70] Greenberg E.D., Gold R., Reichman M., John M., Ivanidze J., Edwards A.M., Johnson C.E., Comunale J.P., Sanelli P. (2010). Diagnostic accuracy of CT angiography and CT perfusion for cerebral vasospasm: A meta-analysis. Am. J. Neuroradiol..

[71] Hansen J.M., Schankin C.J. (2019). Cerebral hemodynamics in the different phases of migraine and cluster headache. J. Cereb. Blood Flow Metab..

[72] Olesen J., Friberg L., Olsen T.S., Iversen H.K., Lassen N.A., Andersen A.R., Karle A. (1990). Timing and topography of cerebral blood flow, aura, and headache during migraine attacks. Ann. Neurol..

[73] Floery D., Vosko M.R., Fellner F.A., Fellner C., Ginthoer C., Gruber F., Ransmayr G., Doerfler A., Uder M., Bradley W.G. (2012). Acute-onset migrainous aura mimicking acute stroke: MR perfusion imaging features. Ajnr Am. J. Neuroradiol..

[74] Linn J., Freilinger T., Morhard D., Brückmann H., Straube A. (2007). Aphasic migraineous aura with left parietal hypoperfusion: A case report. Cephalalgia.

[75] Pollock J.M., Deibler A.R., Burdette J.H., Kraft R.A., Tan H., Evans A.B., Maldjian J.A. (2008). Migraine associated cerebral hyperperfusion with arterial spin-labeled MR imaging. AJNR Am. J. Neuroradiol..

[76] Marchal G., Young A.R., Baron J.C. (1999). Early postischemic hyperperfusion: Pathophysiologic insights from positron emission tomography. J. Cereb. Blood Flow Metab..

[77] Sotoudeh H., Shafaat O., Singhal A., Bag A. (2018). Luxury perfusion: A paradoxical finding and pitfall of CT perfusion in subacute infarction of brain. Radiol. Case Rep..

[78] Yu S., Liebeskind D.S., Dua S., Wilhalme H., Elashoff D., Qiao X.J., Alger J.R., Sanossian N., Starkman S., Ali L.K. (2015). UCLA Stroke Investigators Postischemic hyperperfusion on arterial spin labeled perfusion MRI is linked to hemorrhagic transformation in stroke. J. Cereb. Blood Flow Metab..

[79] Lin Y., Liu H. (2020). Update on cerebral hyperperfusion syndrome. J. Neurointerv. Surg..

[80] Waltz A.G. (1968). Effect of blood pressure on blood flow in ischemic and in nonischemic cerebral cortex. The phenomena of autoregulation and luxury perfusion. Neurology.

[81] Meyers P.M., Higashida R.T., Phatouros C.C., Malek A.M., Lempert T.E., Dowd C.F., Halbach V.V. (2000). Cerebral hyperperfusion syndrome after percutaneous transluminal stenting of the craniocervical arteries. Neurosurgery.

[82] Farooq M.U., Goshgarian C., Min J., Gorelick P.B. (2016). Pathophysiology and management of reperfusion injury and hyperperfusion syndrome after carotid endarterectomy and carotid artery stenting. Exp. Transl. Stroke Med..

[83] Mokin M., Ciambella C.C., Masud M.W., Levy E.I., Snyder K.V., Siddiqui A.H. (2016). Whole-Brain Computed Tomographic Perfusion Imaging in Acute Cerebral Venous Sinus Thrombosis. Interv. Neurol..

[84] Gulati D., Strbian D., Sundararajan S. (2014). Cerebral venous thrombosis: Diagnosis and management. Stroke.

[85] See I., Su J.R., Lale A., Woo E.J., Guh A.Y., Shimabukuro T.T., Streiff M.B., Rao A.K., Wheeler A.P., Beavers S.F. (2021). US Case Reports of Cerebral Venous Sinus Thrombosis With Thrombocytopenia After Ad26.COV2.S Vaccination, March 2 to April 21, 2021. JAMA.

[86] Doege C.A., Tavakolian R., Kerskens C.M., Romero B.I., Lehmann R., Einhäupl K.M., Villringer A. (2001). Perfusion and diffusionmagnetic resonance imaging in human cerebral venous thrombosis. J. Neurol..

[87] Gupta R.K., Bapuraj J.R., Khandelwal N., Khurana D. (2014). Prognostic indices for cerebral venous thrombosis on CT perfusion: A prospective study. Eur. J. Radiol..

[88] Saposnik G., Barinagarrementeria F., Brown R.D., Bushnell C.D., Cucchiara B., Cushman M., deVeber G., Ferro J.M., Tsai F.Y. (2011). American Heart Association Stroke Council and the Council on Epidemiology and Prevention Diagnosis and management of cerebral venous thrombosis: A statement for healthcare professionals from the American Heart Association/American Stroke Association. Stroke.

[89] Van Cauwenberge M.G.A., Dekeyzer S., Nikoubashman O., Dafotakis M., Wiesmann M. (2018). Can perfusion CT unmask postictal stroke mimics? A case-control study of 133 patients. Neurology.

[90] Hauf M., Slotboom J., Nirkko A., von Bredow F., Ozdoba C., Wiest R. (2009). Cortical regional hyperperfusion in nonconvulsive status epilepticus measured by dynamic brain perfusion CT. AJNR Am. J. Neuroradiol..

